# A142 PROGNOSTICATING FACTORS AND OUTCOMES OF EARLY ENDOSCOPIC EVALUATION IN ACUTE UPPER GASTROINTESTINAL BLEEDING

**DOI:** 10.1093/jcag/gwad061.142

**Published:** 2024-02-14

**Authors:** M Saunders, H Melchiorre, L Schmanda, D Savage, P Zezos

**Affiliations:** NOSM University, Sudbury, ON, Canada; NOSM University, Sudbury, ON, Canada; NOSM University, Sudbury, ON, Canada; Emergency Department, Thunder Bay Regional Health Sciences Centre, Thunder Bay, ON, Canada; Division of Gastroenterology, Thunder Bay Regional Health Sciences Centre,, Thunder Bay, ON, Canada

## Abstract

**Background:**

Current guidelines recommend esophagogastroduodenal endoscopy (EGD) early (ampersand:003C24 hours after presentation) for all suspected acute upper gastrointestinal bleeds (AUGIB). Among referral centers providing advanced tiers of care to large regions, treatment is often delayed secondary to geographic constraints. Metropolitan centers meet early EGD recommendations in up to 87.5% of patients. Some centers limit endoscopy to the ICU setting outside of normal hospital operational times. There is currently no data assessing Canadian referral center endoscopic timing, including factors delaying AUGIB endoscopy and outcomes.

**Aims:**

The aim of this study was to report the prevalence of early EGD in AUGIB at a Northern Ontario referral center. Factors associated with delayed EGD and poor outcomes associated with AUGIB were also evaluated.

**Methods:**

Of 441 patients retrospectively identified with an ICD-10 discharge diagnosis of gastrointestinal hemorrhage from 2016–22, 327 patients met inclusion criteria. Patients were excluded if they were below the age of 18 years at the time of presentation, had chronic UGIB, or lower gastrointestinal hemorrhage.

**Results:**

Pre-endoscopic risk stratification via GBS and clinical Rockall score was high at 10 and 3, respectively. There were 279 patients who received early EGD for AUGIB, of which 56% were male. The median (IQR) time to endoscopy was 27.4 (29.3) hours with early EGD achieved in 46% of presentations. A significantly greater proportion of patients presenting to hospital on the weekend (n=105) had delayed EGD (*p*ampersand:003C0.0001; Phi effect size 0.45). Patients presenting at times when EGD was unavailable (n=157) also experienced delayed EGD (*p*ampersand:003C0.05). regional patients did not experience a greater proportion of delayed EGD. In multivariate analysis, presentation on the weekend, higher CCI, absence of pre-existing evidence of liver cirrhosis, and absence of major stigmata of recent hemorrhage on EGD were independent predictors of delayed EGD (Nagelkerke R^2^ = 0.379, *p*ampersand:003C0.0001). The odds ratio for receiving late EGD was 8.9 times greater (95% CI 3.7–21.3) for patients presenting on the weekend (*p*ampersand:003C0.0001). Longer endoscopy wait time was significant correlated with higher pre-EGD blood transfusions and longer length of stay (*p*ampersand:003C0.001). Delayed EGD did not increase all-cause 30-day mortality.

**Conclusions:**

Prevalence of delayed EGD in AUGIB was markedly elevated and associated with hospital presentation during times of limited endoscopic capabilities. Outcomes of delayed GED included increased length of stay and higher blood transfusion rates.

**Funding Agencies:**

NOAMA

